# Emerging role of gut microbiota in autoimmune diseases

**DOI:** 10.3389/fimmu.2024.1365554

**Published:** 2024-05-03

**Authors:** Xinyi Wang, Wei Yuan, Chunjuan Yang, Zhangxue Wang, Jin Zhang, Donghua Xu, Xicai Sun, Wenchang Sun

**Affiliations:** ^1^ School of Clinical and Basic Medical Sciences, Shandong First Medical University & Shandong Academy of Medical Sciences, Jinan, China; ^2^ Department of Radiation Oncology, The First Affiliated Hospital of Shandong Second Medical University, Weifang, China; ^3^ Department of Central Laboratory, The First Affiliated Hospital of Shandong Second Medical University, Weifang, China; ^4^ Department of Rheumatology, The First Affiliated Hospital of Shandong Second Medical University, Weifang, China; ^5^ Department of Hospital Office, The First Affiliated Hospital of Shandong Second Medical University, Weifang, China

**Keywords:** autoimmune disease, homeostasis, gut microbiota, probiotics, microbiome

## Abstract

Accumulating studies have indicated that the gut microbiota plays a pivotal role in the onset of autoimmune diseases by engaging in complex interactions with the host. This review aims to provide a comprehensive overview of the existing literatures concerning the relationship between the gut microbiota and autoimmune diseases, shedding light on the complex interplay between the gut microbiota, the host and the immune system. Furthermore, we aim to summarize the impacts and potential mechanisms that underlie the interactions between the gut microbiota and the host in autoimmune diseases, primarily focusing on systemic lupus erythematosus, rheumatoid arthritis, Sjögren’s syndrome, type 1 diabetes mellitus, ulcerative colitis and psoriasis. The present review will emphasize the clinical significance and potential applications of interventions based on the gut microbiota as innovative adjunctive therapies for autoimmune diseases.

## 1 Introduction

Autoimmune diseases represent a group of chronic and systemic disorders characterized by an excessive immune response, abundant inflammation, and the extensive depositions of immune complexes to tissues and organs. Epidemiological investigations have revealed a global incidence of 5~8% for autoimmune diseases (1, 2). The pathogenesis of autoimmune disease is multifaceted involving genetic predisposition, immune dysregulation, and environmental factors like lifestyle, dietary patterns, and medications (3). Despite advancements in diagnosis and treatment, early diagnosis and precise therapeutic strategies of autoimmune diseases remain significant challenges (4). Increasing evidence has highlighted the pivotal role of the gut microbiota in maintaining immune balance and homeostasis in autoimmune diseases, particularly including rheumatoid arthritis (RA), systemic lupus erythematosus (SLE), systemic scleorosis, and type I diabetes mellitus (T1DM). Disturbances in the composition and diversity of the gut microbiota are strongly associated with autoimmune disorders (5–7). This review aims to elucidate the regulatory mechanisms by which the gut microbiota influence autoimmunity and inflammation, consolidating available evidence on their association with autoimmune diseases. Additionally, it seeks to offer novel insights into early diagnosis and precise treatment strategies for autoimmune diseases.

## 2 Compositions and functions of the gut microbiota

The human gut harbors a diverse array of microbiota, encompassing bacteria, fungi, viruses and assorted microorganisms (8).

With advances in sequencing techniques, such as 16S rRNA and metagenomics, certain intestinal microbiota closely associated with human health have been identified, such as *Phyla Firmicutes*, *Bacteroidetes*, *Pseudomonadota*, and *Actinomycetota*, while others like *Fusobacteria* are relatively less studied (9, 10).

Microbiota populations colonize not only the gut but also the skin, respiratory tract, and reproductive system, influencing various physiological processes, including nutrition, tumorigenesis and immune homeostasis (11). It has been well established that the composition and abundance of the gut microbiota can be influenced by various factors, including environmental factors, diet and the host’s immune system (12, 13). The gut microbiota is not static but dynamic throughout human life. The study by Xie et al. has shown that the gut microbiota diversity changes over time, notably in genetically identical twins living apart (14). Dietary habits also significantly impact microbiota; for instance, high-fiber diets positively correlate with increased abundance of *Lachnospiraceae* (15), while Western diets rich in red meat and low in fiber are associated with *Bacteroides spp* and *Ruminococcus torques* dominance (16). Consequently, microbial compositions vary with dietary habits, heredity and other factors.

The gut microbiota, evolved alongside its host, profoundly influences various physiological and pathological processes, including nutrient production, drug effects, resistance to pathogens, and immune regulation (17–19). Gut bacteria ferment indigestible carbohydrates, generating short-chain fatty acids (SCFAs) like acetate, propionate and butyrate. These SCFAs act as biologically active compounds, providing energy for colonic epithelial cells (20, 21). In particular, butyrate acts as a primary energy source for the colonic epithelial cells (22). Furthermore, the gut microbiota significantly impacts medication metabolism. For instance, certain bacteria convert gemcitabine metabolites and irinotecan HCl, affecting therapeutic efficacy and potential side effects (23). It has been demonstrated that *Escherichia coli (E. coli)*, *Staphylococcus* and *Clostridium sporogenes* produce an enzyme called beta-glucuronidase, converting the harmless form of the chemotherapeutic drug irinotecan HCl (CPT-11), known as SN-38 glucuronide, to its active form, SN-38 (24). Moreover, the gut microbiota exerts crucial effects on the immune system, including impacts on the proliferation and activation of immune cells, autoantibodies generation, and the onset of autoimmune diseases. Studies comparing germ-free (GF) and specific pathogen-free (SPF) mice highlight the microbiota’s impact on innate immune cell modulation and host defense against bacterial infections (25). Additionally, the microbiota contributes to the production of neurochemicals like gamma-aminobutyric acid (GABA), impacting the central nervous system and the gut through the brain-gut axis, ultimately influencing the immune microenvironment (26). Exploring the makeup, diversity, and structural alterations in the body’s microorganisms, along with interactions with the immune system, holds promise for understanding the fundamental processes of autoimmune diseases. All these findings offer the possibilities of identifying new biomarkers and developing effective therapeutic strategies for various diseases.

## 3 Role of microbiota in establishing and maintaining a stable immune system

### 3.1 Microbiota and innate immunity

The correlation between the gut microbiota and innate immunity has attracted significant attention in academic research. The gut-associated lymphoid tissues (GALT) play a crucial role in protecting the intestinal mucosa, working in coordination with the mucosa-associated lymphoid tissues (MALT). Innate immune cells within these tissues employ non-specific pathogen recognition, innate immune reaction initiation and antigens presentation to activate the adaptive immune system (27, 28). It has been shown that the gut microbiota is important in regulating the physiological functions of GALTs in germ-free (GF) models, aiding in their development and maturation (27). Additionally, metabolic by-products produced by commensal microbiota, such as SCFAs, influence the immune response of GALTs through epigenetic mechanisms, supporting the defensive functions and immune tolerance (27).

Innate lymphoid cells (ILCs) are integral to GALTs. The development of ILCs occurs independently of the gut microbiota, while their specific functions rely on commensal microbiota (29, 30). For instance, ILC3, a prominent ILC class, supports epithelial cells survival, antimicrobial peptides production, and the generation of IL-22 (31). IL-22 is a key cytokine essential for the host’s immune response to *Citrobacter* (32, 33). It has been well documented that SCFAs promote IL-22 production of ILC3 by activating aromatic hydrocarbon receptor (AHR) through the AKT-STAT3 and ERK-STAT3 signaling pathways (34, 35) (**Table 1**). Moreover, SCFAs stimulate the proliferation of intestinal ILCs by affecting G protein-coupled receptor (GPCR) activity (45) (**Table 1**). The gut microbiota also facilitates interactions between ILC3 and other cell types (31), promoting the expression of protective proteins like fucosyltransferase 2 (FUT2) that strengthen the intestinal mucosal barrier (60). As the gut microbiota matures, ILC1 levels increase, indicating their reliance on commensal microbiota for development (30).

**Table 1:** Effects of the gut microbiota and microbiota-derived metabolites on the immune system.

Microbiota/Metabolites	Immune cells	Effects/Mechanisms	Ref.
*Lactobacillus sakei K040706*	NK cell	Increasing NK cell activity in the spleen; Promoting the maturation of the spleen germinal center	(36)
*Listeria monocytogenes*	Macrophage	Driving the proliferation of yolk sac-derived macrophages; Influencing the development of stable bone marrow cells; Regulating CCR2; Influencing macrophage homing from the periphery to the gut and development	(37, 38)
*Segmented* *filamentous bacteria*	T cell	Inducing RORγt^+^Th17 cells in GALT	(39)
*Bacteroides fragilis*	T cell	Secreting polysaccharide A; Stimulating IL-10 production by CD4^+^Treg cells	(40)
*Lactobacillus* *Sutterella* *Klebsiella*	B cell	Promoting naïve B cells to differentiate into regulatory B cells in mesenteric lymph nodes; Producing ATP; Activating P2X and P2Y of DC; Inducing IL-6 and TGF-β production; Promoting type arrangement and secretion of IgA	(41)
*Bacillus polyfermenticus*	NK cell	Activating DCs and NK cells; Expanding NK cell pool and increasing cytotoxicity; Amplifying type I response; Promoting IFN-γ secretion by NK cells	(42, 43)
*Lactobacillus* *Bifidobacterium*	T cell	Inducing CD4^+^CD25^+^FoxP3^+^Treg cells	(44)
SCFAs	ILC1	Increasing the number of ILC1	(45)
SCFAs	ILC3	Activating aryl hydrocarbon receptor (AHR); Promoting IL-22 production	(34)
SCFAs	T cell	Regulating the differentiation of CD4^+^T cells; Inducing the generation of Treg cells; Promoting IL-10 production by Th1 cells; Promoting the expression of FOXP3; Negatively regulating the differentiation of Th9 cells; Inhibiting the secretion of IL-9; Inhibiting histone deacetylase activity; Reducing IL-17a secretion	(44, 46–50)
SCFAs		Promoting cell metabolism; Boosting the memory capacity of activated CD8^+^T cells; Regulating the gene expression of CD8^+^T cells and Tc17 cells; Promoting IFN-γ and granzyme B secretion; Promoting molecular switch of Tc17 cells to CTL phenotype	(51, 52)
SCFAs	B cell	Activating intestinal epithelial cells through GPR41 and GPR43 receptors; Promoting the secretion of TSLP and inflammatory factors; Inducing IgA production by B cells; Regulating B cell differentiation; Inhibiting autoantibodies;	(53, 54)
SCFAs	DCs	Inhibiting LPS-induced maturation of DCs Down-regulating the expression of CD80, CD83 and MHC Class II molecules; Enhancing endocytosis; Reducing the release of CCL3, CCL4 and CXCL9; Increasing the expression of IL-10 and IL-23 and inhibiting the production of IL-12 and IFN-γ	(55–57)
SCFAs	Macrophage	Inducing the secretion of NO, IL-6 and IL-12p40 in a dose-dependent manner; Enhancing histone H3 acetylation by acting as an HDAC inhibitor; Promoting the synthesis of the anti-inflammatory cytokine IL-10	(58, 59)

Conventional natural killer (NK) cells, the sole cytotoxic cell population within innate immunity, detect pathogens and carry out cytotoxic functions by releasing proteins like granzymes (61). The precise mechanisms regulating NK cell equilibrium between alloreactivity and autotolerance remain unclear. The gut microbiota, containing ligands for NK cell receptors, influences NK cell function and cytotoxicity, as observed in GF mice with the absence of interaction with commensal bacteria.

Macrophages in the gut are crucial for defending against infections and play a pivotal role in maintaining the integrity of the intestinal mucosa. The gut microbiota influences the development of myeloid-derived macrophages and the intestinal inflammation by modulating myeloid cell hematopoiesis, migration, and population sustenance within the gut (37, 38, 62–64) (**Table 1**).The relationship between the gut microbiota and innate immune cells underscores the profound influence of microbial communities on the development, differentiation, and functionality of these vital immune components, thus significantly contributing to the gut’ s microecology balance.

The intricate interplay between the gut microbiota and the local innate immune system contributes to the shaping of the gut microenvironment. Increasing evidence has demonstrated that the dysregulation of gut microbiota can result in disruptions and imbalance of the innate immune system by regulating the TLR signaling activation, inflammasome response, and ILC alterations, ultimately contributing to the onset of autoimmune diseases, such as RA and SLE (27, 65). Furthermore, fecal transplantation from healthy mice to certain disease-model mice has been shown to alleviate symptoms and ameliorate metabolic irregularities due to impaired activation of innate immune receptors, such as type 1 diabetes mellitus (T1DM) and inflammatory bowel disease (IBD) (31, 66). These findings have suggested the crucial role of gut microbiota in regulating autoimmune disorders through influencing innate immunity.

### 3.2 Microbiota and adaptive immunity

The gut microbiota plays a pivotal role in establishing and sustaining adaptive immunity in the gut, orchestrating interactions between varieties of immune cell types like T cells and B cells to ensure immunological equilibrium. T cells within the gut, specifically the CD4^+^T cells known as Th cells, display remarkable diversity and functions shaped by the unique metabolic characteristics of the gut microbiota (67). Notably, the gut microbiota influences the differentiation of naïve CD4^+^T cells into subsets like Th17 cells and Treg cells. Th17 cells are vital in defending against bacterial and fungal infections in the lamina propria (LP) of the small intestine by producing IL-17A, IL-17F, and IL-22. They also promote intestinal epithelial cells for more production of antimicrobial peptides (AMP), activate endothelial cells (ECs), and aid in neutrophils recruitment (68, 69). *Segmented filamentous bacteria* (SFB), a relatively low-abundance microbial population in the ileum, can induce the generation of RORγt^+^Th17 cells in the tissue-associated lymph nodes in the gut, although the excessive activation of RORγt^+^Th17 cells might lead to autoimmune diseases (70).

In contrast, regulatory T (Treg) cells contribute to immunological tolerance by promoting self-tolerance and suppressing excessive immune activation (71). The forkhead box P3 (Foxp3) is the key transcription factor for CD4^+^CD25^+^Treg cells, inhibiting excessive immune reactions. Certain probiotics, like *Lactobacillus* and *Bifidobacterium infantis* induce the production of anti-inflammatory CD4^+^CD25^+^Foxp3^+^ Treg cells, while *Bacteroides fragilis* and its polysaccharide A (PSA) stimulate IL-10 production by CD4^+^Treg cells depending on IL-2 pathway, suppressing inflammation (72). In addition, some bacterial metabolites, such as adenosine and inosine, can interact with T cells’ adenosine A2A receptor (A2AR), enhancing Treg cell activity while inhibiting Th1 and Th17 inflammatory responses (73).

**Figure 1:**
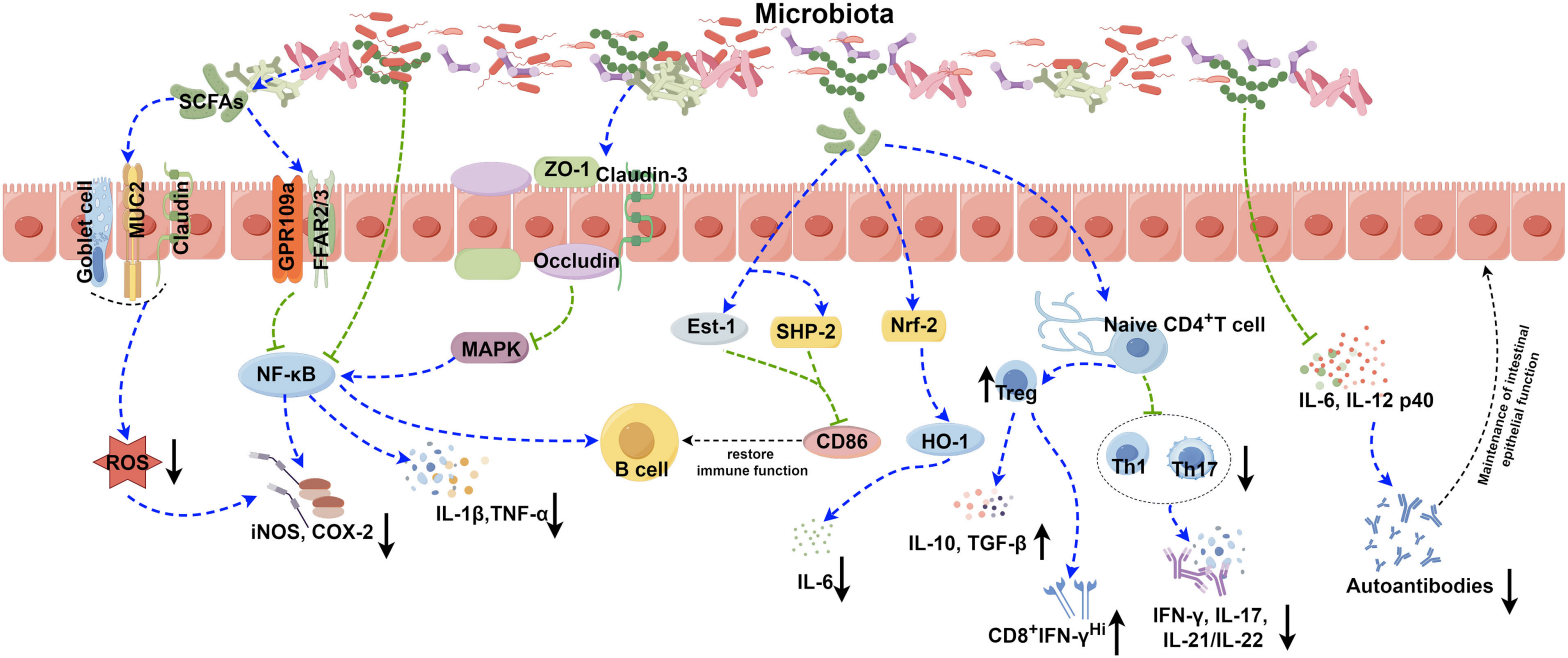
Role of the gut microbiota in autoimmune disease. The gut microbiota contributes to the pathogenesis of autoimmune diseases through various complicated mechanisms, such as the secretion of SCFAs, modulation of NF-κB and Nrf2 signaling pathways, disruption of the balance between Treg, Th1, and Th17 cells, and modulation the release of inflammatory factors. The image was created utilizing the FigDraw online platform (https://www.figdraw.com/#/) and was identified by the in-house image ID: UPORSc4cf4. SCFAs: Short chain fatty acids; MUC2: Recombinant Mucin 2; ROS: Reactive oxygen species; GPR109a: G protein-coupled receptor 109a; FFAR2/3: Recombinant Free Fatty Acid Receptor 2/3; NF-κB: Nuclear factor kappa-B; COX-2: Cyclooxygenase 2; iNOS: Inducible nitric oxide synthase; ZO-1: Zona Occludens 1; MAPK: Mitogen-activated protein kinase; IL-1β: Interleukin-1 beta; TNF-α: Tumor necrosis factor alpha; Est-1: Estrogen sulfotransferase-1; SHP-2: SH2 domain-containing protein-tyrosine phosphatase-2; Nrf-2: NF-E2-related factor 2; HO-1: Recombinant Heme Oxygenase 1; IL-6: Interleukin-6; IL-10: Interleukin-10; TGF-β: Transforming growth factor beta; IFN-γ: Interferon gamma; IL-17: Interleukin-17; IL-21/IL-22: Interleukin-21/Interleukin-22; IL-12 p40: Interleukin-12 p40.

Several pathways, including B cell receptor (BCR), CD40, Toll-like receptors (TLRs), B cell activating factor receptor (BAFF), and proliferation-inducing ligand (APRIL) receptors, primarily control the activation and differentiation of B cells (74). Activation of TLRs and BAFF/APRIL receptors significantly impacts T cell-dependent B cell antibody production (74). It is firmly established that the gut microbiota modulates B cell function by interacting with BCR through antigenic determinants and activating TLRs and NOD-like receptors (NLRs) via specific metabolites (74). Moreover, the gut microbiota stimulates dendritic cells (DCs) and the intestinal tissue cells to release cytokines like IL-1β and IL-6, which enhances the differentiation of naïve B cells into regulatory B cells (Bregs) within mesenteric lymph nodes (MLN) (41) (**Table 1**). Additionally, microbiota-derived ATP in the gut is converted to adenosine, activating adenosine receptors on B cells and promoting the production of IgG and IgA antibodies (74). Beyond these direct effects, the gut microbiota indirectly influences B cell differentiation and activation. As gut microbiota-derived metabolites, SCFAs can activate the intestinal epithelial cells via the cell receptors, such as GPR41, GPR43, and FFAR2, which prompts the release of thymic stromal lymphopoietin (TSLP) by epithelial cells and the release of TNF-α and iNOS of dendritic cells. This cascade triggers the expression of APRIL and IL-10, further enhancing IgA antibody production by B cells (75). Additionally, SCFAs can also activate receptors on the intestinal epithelial cells, like FFAR2, FFAR3 or GPR109a, and modulate the nuclear factor-κ-light chain enhancer (NF-κB) pathway involved in B cell activation, thereby suppressing inflammatory responses (**Figure 1**). Consequently, the gut microbiota and its metabolites are closely intertwined with the development, differentiation, and activation of B cells, exerting a substantial influence on immune responses and inflammation.

It has been well documented certain gut microorganisms harboring epitopes resembling host proteins, such as the RNA-binding protein Ro60, are capable of activating T and B cells in SLE, thereby triggering abnormal adaptive immune response and inducing production of pathogenic autoantibodies (76). Besides, the roles of Th9 cells and IL-9 have been demonstrated in UC, functioning to impair intestinal barrier function, prevent mucosal wound healing *in vivo* and compromise tolerance to commensal bacteria by inducing inflammation and adaptive immune disorders (77–79). Therefore, the gut microbiota may contribute to autoimmune diseases by regulating T- or B-cell mediated adaptive immunity. The complex interplay between the gut microbiota and immune cells influences immune responses and homeostasis. Metabolites like SCFAs significantly impact cytokine and immunoglobulin production, affecting the progression of autoimmune diseases. This close interaction between the microbiota and the immune system forms a protective barrier against various threats. Alterations in the microbiota can lead to immune dysfunction and the onset of autoimmune diseases.

## 4 Role of microbiota and autoimmune diseases

The integrity of human gut microbiota has been shown to correlate with susceptibility and outcomes of various diseases, such as metabolic, infectious and autoimmune diseases (80). As suggested in a groundbreaking article in 2002, the incidences of some classic infectious diseases such as tuberculosis and measles as well as intestinal infections have declined significantly but with a higher incidence of autoimmune diseases like T1DM and asthma in western countries in the last 50 years of the 20th century due to the improved sanitation, antibiotics usage and vaccination (81). This trend of change could still be observed in the last few years even until now that the incidence of autoimmune diseases is still steadily increasing, accompanied by a steady decline in the incidence of primary infectious diseases (82). According to “old friend hypothesis”, limited exposure to specific microbes (“old friends”) prevent immune system from forming a tolerogenic microenvironment, especially in early childhood phase (83–86). For children, the delivery mode, diet, and exposure to antibiotics and antacids are common factors for the contact with microbiota. Researches have shown that infants undergoing vaginal delivery and exclusive breastmilk feeding usually have a lower cumulative allergic burden, while those who exposed to antibiotic and antacid have an increased cumulative allergic burden conversely, thereby demonstrating the importance of microbiota in resisting excessive autoimmune response (87). Therefore, investigating the development, management, and prognosis of autoimmune disorders holds paramount importance due to the potential impact of microbiota dysfunction on both immunodeficiency syndromes and autoimmune diseases.

Dysbacteriosis, a commonly recognized term, refers to disruptions in the composition or activity of the microbiota within specific anatomical areas. This disruption encompasses alterations in both α-diversity and β-diversity. α-diversity reflects the variability in types and quantities of microorganisms of the host, while β-diversity delineates differences in microbial community makeup between individuals (88). Recent research has increasingly linked microbiota dysregulation to compromised intestinal barrier integrity, diminished functionality, and enhanced inflammation in autoimmune diseases (89, 90). Therefore, a growing body of evidence has underscored the intimate relationship between microbiota compositions and these conditions.

### 4.1 Microbiota and systemic lupus erythematosus

SLE is one of the most prevalent autoimmune diseases with multisystemic clinical manifestations caused by abundant immune complex depositions to tissues and target organs, leading to long sustained inflammation, immune disorders and ultimately multi-organ damages (91). A previous study has suggested that transferring microbiota from the cecum of lupus-prone mice into healthy mice can induce lupus (92). Similarly, GF mice receiving fecal matter from lupus mice exhibit increased levels of anti-DsDNA antibodies in serum, implicating the crucial role of the gut microbiota in autoantibody formation and immune reactions (93). Consequently, the gut microbiota is recognized as a pivotal factor contributing to SLE (27, 94, 95). The migration of microbial products from the colon and elevated permeability of the intestinal mucosal barrier are well recognized mechanisms implicated in the initiation and progression of SLE. Research in lupus-susceptible (NZB×BXSB) F1 mice has shown the migration of *Enterococcus gallinarum* from the intestine to mesenteric veins, intestinal draining lymph nodes, liver, and spleen (96). *Enterococcus gallinarum* has been also detected in liver biopsy specimens from SLE patients (96). Calprotectin, a calcium-containing protein in neutrophils and macrophages, acts as a crucial biomarker for impaired intestinal barrier (97). A close association between damaged intestinal barrier function in SLE patients and elevated levels of fecal calprotectin has been well documented (96, 98). Furthermore, certain gut microorganisms harboring epitopes resembling host proteins are capable of activating T and B cells, thereby triggering abnormal immune responses and abundant production of pathogenic autoantibodies. For instance, the RNA-binding protein Ro60, found in various gut microorganisms, shares homologous sequences with human Ro60 epitopes (76, 99). Anti-Ro60 antibodies can induce the generation of autoantibodies against Ro52, Smith, or U1RNP by spreading their epitopes (76, 99). Therefore, the intestinal microbiota containing sequences akin to human Ro60 epitopes may contribute to SLE by exacerbating pathological damages and dysregulated autoimmune responses in lupus-susceptible individuals.

It has been reported that certain gut microbiota can exert inhibitory effects on SLE through various mechanisms (**Figure 1**). *Lactobacillus* has been demonstrated to possess the capability to diminish the number of ILC3 cells and Th17 cells, suppress the pro-inflammatory factor IL-17 production, shift the Treg/Th17 balance in favor of the Treg phenotype, stimulate IL-10 production, and limit the accumulation of IgG-2a in the kidney (94, 100). This cascade ultimately reduces the kidney injures induced by dysregulated autoimmune responses. Moreover, it has been reported that *Lactobacillus* can significantly enhance the expression of key molecules associated with the intestinal mucosal barrier, such as ZO1, occludin and Cldn1 (100). *Lactobacillus* helps to enhance the intestinal barrier’s functionality without affecting the expression of Cldn2, responsible for creating lining pores (100) (**Table 2**). MicroRNAs (miRNAs) play pivotal roles in diverse biological processes, including immune cell maturation, the establishment of central and peripheral tolerance, and the differentiation of T helper (Th) cells. Alterations in miRNA expression can lead to immune system dysfunctions (123). Earlier studies have indicated a positive correlation between elevated expression levels of miR-155 and miR-181a and increased disease severity of SLE patients. Conversely, miR-155 deficiency reduces anti-dsDNA IgG titers and alleviates disease symptoms (124, 125). Interestingly, it has been discovered that *Lactobacillus rhamnosus* and *Lactobacillus delbrueckii* can attenuate the activity of miR-155 and miR-181a in peripheral blood mononuclear cells (PBMCs) of SLE patients (126). However, it’s noteworthy that *Lactobacillus reuteri* can exacerbate SLE by increasing the expression of type I interferon gene in the spleen and ileum of C57/B6 mice, resulting in anemia, increased intestinal permeability, and immune dysfunctions (127). These findings have suggested that different *Lactobacillus* strains may induce distinct immune responses in varying conditions in mice, leading to diverse outcomes (128). Additionally, the *Firmicutes*/*Bacteroidetes* (F/B) ratio is found to be significantly lower in SLE patients compared to non-SLE individuals (129–131), with *Firmicutes* showing a negative correlation with SLE Disease Activity Index (SLEDAI) scores (132). This suggests that *Firmicutes* may potentially delay the progression of SLE. Butyric acid and propionic acid, produced by *Firmicutes*, directly impact B cells by promoting the differentiation and proliferation of extrathymic Treg cells. Additionally, they suppress the expression of LPS-induced inflammatory cytokines such as IL-6, IL-12, and p40, thereby reducing the production of autoantibodies. Furthermore, these acids enhance and sustain the integrity of the intestinal epithelial barrier function in lupus-prone animals (57, 133, 134).

**Table 2:** Roles and mechanisms of probiotics in regulating autoimmune diseases.

Disease	Probiotics/Metabolites	Effect/Mechanism	Ref.
SLE	*Lactobacillus*	Increasing the secretion of IL-10; Inhibiting the secretion of IL-17; Increasing the production of ZO-1, occludin and Cldn1; Enhancing intestinal barrier function	(100)
SLE	*Bifidobacterium* *Lactobacillus*	Binding to symbiotic bacteria; ActivatingFFAR2, FFAR3 or GPR109a; Inhibiting inflammatory response; Blocking nuclear factor-κ light chain enhancers of the B-cell activation	(73)
SLE	SCFAs	Inhibiting IL-6, IL-12 and p40; Decreasing autoantibody production; Maintaining intestinal epithelial barrier function	(57)
SLE	*Bacteroides fragilis*	Increasing CD1d expression via Est-1 signaling pathway in B cells; Inhibiting CD86 expression via SHP-2 signaling pathway; Restoring the immune response of B cells	(101)
RA	*Prevotella histicola*	Increasing the number of Treg cells; Inhibiting the response of Th17; Promoting the release of IL-10; Increasing the expression of ZO-1 and occluding; Maintaining intestinal barrier function;	(102)
RA	*Lactobacillus casei*	Reducing joint swelling; Inhibiting RA pathophysiology; Decreasing arthritis score; Reducing inflammatory cytokines; Decreasing total cholesterol and low-density lipoprotein cholesterol levels in blood	(103)
RA	*Bifidobacterium adolescentis*	Competing for growth factors, Reducing vitamin K; Inhibiting *Porphyromonas gingivalis* growth	(104)
RA	*Faecalibacterium prausnitzii*	Increasing butyrate production; Promoting IL-10 secretion	(105)
SS	*Enterococcus faecalis* *Saccharomyces boulardii*	Alleviating subjective symptoms; Increasing secretion of tears; Prolonging tear film breakup time	(106, 107)
SS	*Bifidobacterium*	Resisting pathogens; Reducing bacterial growth in the tear film	(108)
SS	*Lactobacillus casei* *Lactobacillus acidophilus* *Lactobacillus reuteri* *Bifidobacterium* *Streptococcus thermophilus*	Decreasing the number of CD8^+^ IFN-γ^Hi^ cells; Increasing the number of Treg cells	(109)
T1DM	*Bifidobacterium lactis HY8101*	Up-regulating GLUT4 and PPAR-γ in TNF-α-treated L6 cells; Down-regulating PCK1 and G6PC; Decreasing fasting insulin and blood glucose; Improving insulin tolerance; Decreasing plasma total cholesterol and triglyceride levels	(110)
T1DM	*L. johnsonii N6.2*	Increasing the expression of Claudin-1; Decreasing the expression of occludin; Increasing the number of goblet cells; Promoting the formation of an anti-inflammatory environment; Down-regulating iNOS and IFNγ; Decreasing mature caspase-1	(111, 112)
T1DM	*Lactobacillus* *Bifidobacterium*	Promoting the release of GLP-1, Resulting in reduced food intake and improved glucose tolerance; Increasing the levels of butyrate	(113)
T1DM	*Lactobacillus salivarius subsp. salicinius AP-32* *L. johnsonii MH-68* *Bifidobacterium animalis subsp. lactis CP-9*	Decreasing fasting blood glucose and HbA1c levels; Reducing the levels of inflammatory cytokines; Increasing the expression of TGF-β1	(114)
T1DM	*Saccharomyces boulardii Tht 500101*	Increasing the C-peptide levels and hepatic glycogen content to lower glycemia; Regulating fat metabolism; Promoting the recovery of the gut microbiota	(115)
UC	*Lactobacillus plantarum SC-5*	Inhibiting activation of MAPK and negatively regulating NF-κB; Increasing the expression of ZO-1, Occludin, and Claudin-3; Regulating the balance of the gut microbiota	(116)
UC	*Bifidobacterium longum subsp. longum YS108R*	Inhibiting NF-κB signaling pathway; Activating Nrf2 signaling pathway; Increasing SCFA-producing bacteria and declining Gram-negative bacteria	(117)
UC	*Limosilactobacillus mucosae CCFM1273*	Mitigating the disease symptoms and colonic pathologic damage; Restoring goblet cell numbers and MUC2 production; Enhancing intercellular junctions; Inhibiting Fas/Fasl pathway and epithelial cell apoptosis; Inhibiting NF-κB signaling pathway; Increasing SCFA-producing bacteria and declining Gram-negative bacteria	(118)
UC	*Lacticaseibacillus rhamnosus 2016SWU.05.0601*	Reducing the expression of inflammatory cytokines; Regulating the activation of NF-κB-iNOS/COX-2 signaling pathway	(119)
psoriasis	*Bifidobacterium infantis strain 35624*	Decreasing the levels of CRP and TNF-α	(120)
psoriasis	*Lactobacillus*	Decreasing hs-CRP and MDA levels and increasing total antioxidant capacity; Alleviating disease symptoms	(121)
psoriasis	*Bacillus* spp.	Improving quality of life with lower PASI and DLQI scores; Reducing the levels of TNFα, IL-6, and IFN-γ, but enhancing the levels of IL-10; Enhancing the diversity of the gut microbiota	(122)

### 4.2 Microbiota and rheumatoid arthritis

The gut microbiota composition in early RA patients differs significantly from that of healthy individuals, characterized by a significant reduction in *Bifidobacterium* and *Bacteroides* families and a notable increase in *Prevotella* species (135–137). These distinctions suggest a potential contribution of the gut microbiota-host interaction to the onset and progression of RA. However, the precise mechanism remains unclear. Recent studies propose that the influence of the gut microbiota on RA might involve various mechanisms, including the activation of antigen-presenting cells, production of citrullinated peptides through interactions with Toll-like receptors (TLRs) or NOD-like receptors (NLRs), induction of antigen-mimicking cross-reactivity, alterations in the intestinal mucosal permeability, and the promotion of Th17 cell-mediated inflammation in the mucosa (138, 139) (**Figure 1**).

Citrullinated peptides, formed when arginine residues are converted into citrullinated residues by protein arginine deiminase (PAD), can disrupt immune tolerance and trigger autoimmune reactions in individuals genetically predisposed to RA under specific conditions (140–142). The gut microbiota dysregulation may compromise PAD function, impacting immunological tolerance and contributing to autoimmune diseases (138, 143–145). Additionally, the gut microbiota itself can encode bacterial PAD enzymes, facilitating citrullination (146, 147). Different species of *Prevotella* play varying roles in RA. Overcolonization of *Prevotella copri* (*P. copri*) might intensify mucosal inflammation and induce immune responses, potentially leading to arthritis (138). *P. copri* can also compromise the intestinal barrier integrity by disrupting the tight junctions (TJs) between intestinal epithelial cells, facilitating disease occurrence (139, 148). Conversely, Marietta et al. have demonstrated that *Prevotella histicola* (*P. histicola*) can prevent and treat collagen-induced arthritis (CIA) in HLA-DQ8 transgenic mice by boosting Treg cells, suppressing Th17 responses, enhancing IL-10 release, and stabilizing the intestinal barrier (102) (**Table 2**, **Figure 1**).

Various strains of *Lactobacillus casei (L. casei)* in preclinical studies have shown efficacy in treating RA, including reducing joint swelling, arthritis scores, and serum inflammatory cytokine levels, highlighting probiotics’ potential in RA remission (103, 149, 150). Additionally, the interplay between periodontal disease and RA involves shared pathogenic mechanisms and immunological pathways. RA patients often display elevated antibody levels against *Porphyromonas gingivalis (P. gingivalis)* and *Prevotella*, with *P. gingivalis* antibodies correlating with the levels of RA-specific anti-CCP antibodies (151, 152).

The enzyme PAD from *P. gingivalis* in periodontal disease can citrullinate human fibrinogen and α-enolase. Antibodies generated against these citrullinated antigens may cross-react with joint antigens, exacerbating RA-related inflammation (153). Researchers are actively exploring strategies to mitigate microbial self-antigen cross-reactivity and curb excessive citrullination, aiming to develop microbiota-based treatments for RA.

### 4.3 Microbiota and Sjögren syndrome

SS is one of the most prevalent autoimmune diseases, primarily affecting the lacrimal and salivary glands. The precise pathogenesis of SS remains largely unknown. Dysfunction of T cells and B cells play a critical role in the onset and progression of SS (154, 155). An imbalance, marked by an increase in Th17 cells and a decrease in Treg cells, can prompt lymphocyte infiltration, epithelial cell activation, enhanced proinflammatory cytokines production (e.g., IFN-γ and IL-17), exposure to autoantibodies, and damages to the corneal barrier, contributing to the development of SS (156–158). Furthermore, Th1 cells, known contributors to the pathogenesis of SS, partake in ocular inflammation by secreting pro-inflammatory cytokines like IFN-γ, IL-1β, IL-6 and TNF-α (159). Elevated expression of B cell activating factor (BAFF) is mainly induced by type I and type II interferons (IFN) (160). Significantly elevated levels of BAFF in the peripheral circulation and the salivary gland tissues have been observed in 55% of SS patients, highlighting increased B cell activation in SS (154, 161). A significant reduction in the diversity of the gut microbiota has been reported in SS patients, characterized by diminished symbiotic bacteria and increased potentially pathogenic strains, positively correlated with disease severity (158). Fecal transplantation (FMT) effectively ameliorates ocular symptoms in germ-free (GF) mice with SS, highlighting the strong link between gut microbiota and SS pathogenesis (162–164). The protective role of the gut microbiota in SS operates through two main mechanisms (**Figure 1**). Firstly, microbiota-produced metabolites like SCFAs exert anti-inflammatory properties, alleviating ocular inflammation (165–167). Secondly, the gut microbiota plays a critical role in regulating the development, differentiation and activation of ocular immune cells, effectively modulating the balance between pro-inflammatory Th17 cells and anti-inflammatory Treg cells (109, 165, 168). As a result, strategies aiming at maintaining microbial balance may hold promise for effective biological prevention approaches for SS.

### 4.4 Microbiota and T1DM

T1DM emerges from a multifaceted autoimmune process, characterized by the T-cell-mediated destruction of insulin-producing β-cells. It is the most commonly diagnosed diabetes in young population, while the incidence of T1DM in adult has also increased rapidly in the past 15 years (169, 170). More and more evidence provide supports that the composition of the gut microbiota, as an environment factor, is different between diabetes patients and healthy individuals. *Bacteroidetes* has been recognized as the most common microbial phylum in T1DM patients, while the number of butyrate-producing species from *Clostridium clusters IV* and *XIVa*, as well as mucin-degrading bacteria such as *Prevotella* and *Akkermansia* in T1DM patients has significantly reduced (171). The importance of butyrate in preserving the integrity of the intestinal mucosal barrier has been highlighted (172) (**Figure 1**). The reduction in butyrate-producing bacteria leads to increased gut permeability. And it will allow the passage of microbial antigens, products, and even the microorganisms themselves, which may promote the inflammation and the progression of T1DM (173, 174). In addition to this, there are many kinds of microbiota that can have an impact on the development of disease. An increase in the gut microbiota and abundance of *Firmicutes* was observed in non-obese diabetic (NOD) mice fed with human lacto-oligosaccharide, helping to inhibit islet inflammation and diabetes (175). Although *Prevotella* has been associated with the development of chronic inflammatory diseases by augmenting mucosal Th17-mediated immune responses, it has also been established that *Prevotella* play a role in protecting against Bacteroides-induced glucose intolerance, promoting glycogen storage, and enhancing glucose metabolism (176, 177). When transplanting human amniotic mesenchymal stem cells (hAMSC) into T1DM mice, scientists noticed that the therapeutic effect of MSC strongly depends on the modification of the beneficial gut microbiota, including *Bifidobacterium*, *Provi­dencia*, *Veillonella*, and *Prevotella*, indicating the significant role of the gut microbiota in alleviating symptom and controlling the development of T1DM (178). Except for forming the intestinal mucosal barrier, the metabolic products of the gut microbiota, such as tryptophan derivatives and SCFAs, also regulates intestinal immunity. SCFAs protect NOD mice from insulitis and slow down the development of T1DM by inhibiting inflammatory responses and the accumulation of IFN-γ^+^T cell in the pancreas (179, 180). As the precursor of SCFAs and an important by-product, intracellular succinic acid could activate intestinal gluconeogenesis positively to regulate gluconeogenesis and blood glucose levels (181, 182). Hence, clinical treatments based on the gut microbiota show promise in maintaining intestinal homeostasis, slow down disease progression, and even reverse T1DM.

### 4.5 Microbiota and ulcerative colitis

UC is a kind of IBD characterized by unpredictable and chronic clinical symptoms, with alternating periods of exacerbation and remission (183). Based on next-generation sequencing technique, scientists have found reduced bacterial diversity and imbalance between beneficial and aggressive bacteria in UC cases (184). Species from the *Proteobacteria* including *E. coli*, *Enterobacteriaceae*, *Klebsiella*, and *Proteus* spp., as well as members from *Fusobacteria*, could enhance inflammatory response and aggravate symptoms, and have been demonstrated to be positively associated with UC (185–188). The microbial metabolites also vary between UC patients and the healthy. SCFAs plays an important role in suppressing intestinal inflammation (34, 189). SCFAs could also induce the production of T-cell-dependent IgA and maintain mucosal homeostasis by regulating the localization of commensal bacteria (190). It has been determined that acetic acid and butyric acid in feces of UC patients were lower than those in the healthy individuals (191). Taken together, all of the changes reveal the close relationships between the gut microbiota and the development of UC (**Figure 1**). However, given the individual phenotypic differences in the gut microbiota, it is still necessary to further study the exact effect mechanisms between individual microbiota and the host (192).

### 4.6 Microbiota and psoriasis

The pathogenesis of psoriasis is complex and not very clear. For now, Th17/IL-23 axis has been established as a crucial immunological mechanism in the development of psoriasis, which is also the basis of biologics treatment (193–195). The gut microbiota and their metabolites could adjust the balance between immune tolerance and inflammation, such as acting on differentiation of naïve T cells into either regulatory or Th17 lineages, so that affecting the progression of psoriasis (168) (**Figure 1**). It has been found that the gut microbiota has impact on the manifestation of the psoriatic phenotype through a Th17-mediated T-cell response on imiquimod-induced mouse models; meanwhile, germ-free mice or conventionally housed mice treated with antibiotics showed protective effect on skin (196) (**Figure 1**). Scientists also noticed an interesting phenomenon that the gut microbiota dysbiosis in psoriasis patients is similar to those of IBD, both with reduced *Eubacterium rectale (E. rectale)*, *Alistipes finegoldii (A. finegoldii)* and *Alistipes shahii (A. shahii)* species (197–200). Based on this consistency, using probiotics to restore the gut microbiota homeostasis and reduce inflammation to achieve therapeutic purpose for psoriasis is possible. Probiotic could suppress the expression of TNF−α, IL−6 and proinflammatory cytokines in the IL−23/IL−17 cytokine axis and enhance gut barrier function to prevent further infection (201). In another case, patients with severe pustular psoriasis showed obvious clinical improvement within 2 weeks after applying *Lactobacillus sporogenes* supplementation 3 times per day, and almost get remission after 4 weeks (202).

In this manuscript, our focus was directed towards examining the involvement of certain prevalent autoimmune diseases of the gut microbiota, including SLE, RA, SS, T1DM, UC, and psoriasis. However, diseases such as multiple sclerosis (MS), autoimmune thyroid disease (AITD), celiac disease (CeD), among others, were not addressed. Further research is warranted to explore the underlying mechanisms governing immune disorders.

## 5 Conclusions and prospects

The interaction between human microbiota and the host plays a crucial role in maintaining health and influencing disease onset. The complex relationship encompasses multiple facets, with microbiota and their metabolites wielding significant influence over host inflammation and immune responses. The gut microbiota participates in regulating immune cell proliferation, differentiation, activation, intestinal permeability, and the integrity of mucosal barriers. Probiotics have emerged as a promising strategy for managing autoimmune diseases, such as SLE and RA. They operate by promoting a healthy gut microbiota and fostering a balanced interaction with the host’s immune system. However, further investigations are warranted to identify specific biomarkers that can accurately distinguish between healthy and compromised microbiota states. Additionally, understanding how microbiota and their metabolites impact normal balanced states versus inflammatory conditions, and discerning potential differences between effects on mucosal surfaces and systemic tissues, remains crucial. In-depth studies investigating the role of microbiota in autoimmune diseases provide insights into the underlying mechanisms of diseases. These insights may reveal prominent diagnostic markers and therapeutic targets, ultimately help us to understand the pathogenesis of autoimmune diseases and explore novel diagnostic and therapeutic strategies.

## Author contributions

XW: Writing – original draft. WY: Writing – review & editing. CY: Writing – review & editing. ZW: Writing – review & editing. JZ: Writing – review & editing. DX: Writing – review & editing. XS: Writing – review & editing. WS: Writing – review & editing.
